# Association Between Hormonal Birth Control, Substance Use, and Depression

**DOI:** 10.3389/fpsyt.2022.772412

**Published:** 2022-02-08

**Authors:** Sharlene D. Newman

**Affiliations:** Department of Psychology, College of Arts and Sciences, University of Alabama, Tuscaloosa, AL, United States

**Keywords:** depression, birth control, alcohol, cannabis, hormones

## Abstract

**Objective:**

The current study examined the impact of the use of hormonal birth control, cannabis (CB), and alcohol on depression symptoms.

**Study Design:**

Survey data from 3,320 college-aged women collected over a 2-year period. Depression symptoms were assessed using the PHQ-9.

**Results:**

Individuals taking hormonal birth control (*N* = 998; age = 19.1 ± 1.6 years) had lower overall depression scores than did those not taking birth control (*N* = 2,322; age = 19.1 ± 1.8 years) with 15.2% of those not taking hormonal birth control had depressive symptoms while 12.1% of those in the birth control group had depressive symptoms. Additionally, those taking hormonal birth control had higher scores on the alcohol and CB use assessment. A between-subjects ANOVA with depression score as the dependent variable found significant effects hormonal birth control use, CB and alcohol use, as well as a significant interaction between CB use and hormonal birth control use.

**Conclusions:**

While there are some limitations (e.g., the between subjects design makes it such that there may be uncontrolled differences between groups), the results suggest that hormonal birth control use may help to reduce depressive symptoms.

**Implications:**

More studies examining the impact of hormonal birth control and substance use on depression are required. The results suggest a potential interaction between CB and hormonal birth control use on depression symptoms that is not observed for alcohol. This implies that alcohol and CB may be linked to depression via different mechanisms.

## Highlights

- Alcohol, cannabis and hormonal birth control use all associated with PHQ-9 depression score.
- Hormonal birth control use and cannabis user interact.
- There is no observed interaction between hormonal birth control and alcohol use.

## Introduction

The college-aged, emerging adult developmental stage is one of great change that coincides with widespread alterations in synaptic connectivity and white matter myelination, including in the prefrontal cortex (1). The emerging adult developmental stage is one in which individuals may experience their first episode of psychiatric disorders such as depression and anxiety with 30% of college students reporting having depressive symptoms (2). Additionally, emerging adulthood is characterized by an increase in engagement in risk-taking behavior including the use of alcohol and cannabis (CB) (3, 4). The growing research on sex differences has also revealed significant sex differences in both depression prevalence and the effect of CB and alcohol use. In fact, clinical and preclinical studies show that females have greater vulnerability toward drug abuse at all stages of the addiction cycle including drug initiation, binging, withdrawal, and relapse (5, 6). Also, female substance users show an increase in sexual risk-taking (7); and an increased risk of depression and anxiety (8, 9). These sex differences in the effects of substance use and the incidence of depression are both thought to be influenced by sex hormones.

Not only is depression more prevalant in emerging adult women, the lifetime prevalence of depression is significantly higher in women compared to men (10–15). Sex hormones are a likely cause of many cases of depression in women (16, 17). For example, oestrogens have been shown to play a protective role in modulating serotonin which has important implications for mood disorders (18, 19). Furthermore, a subtype of depression, referred to as reproductive depression, occurs during hormonal change including during the premenstrual phase of the menstrual cycle, postnatally, and during the transition to men-pause (17, 20) suggesting a relationship between hormonal fluctuations and depression. It should be noted that even though these later in life hormonal changes have been linked to depression, a 2003 study by Kessler found that sex differences in depression prevalence is due primarily to differences at depression onset during adolescence and emerging adulthood, not to later life experiences demonstrating the importance of examining this emerging adulthood developmental period.

In addition to sex hormones, substance use has also been associated with depression (21) with one-third of people with depression meeting criteria for an alcohol use disorder (22). CB use, particularly heavy use, has also been linked to psychiatric disorders (23, 24). For example, a meta- analysis of longitudinal studies examining the relationship between CB use and depression found a moderate association between heavy CB use (defined as at least weekly use) and increased risk of developing depression (23). There is also an association between alcohol use disorder and depression (25) with some suggestion of a causal link with increasing alcohol use resulting in increased risk of depression (26). Interestingly, relationships between sex hormones and substance use have also been observed. For example, there is evidence of increased alcohol (27) and CB (28) use during the premenstrual phase suggesting a link between substance use, depression and fluctuating sex hormones. There is also a growing literature that demonstrates that estrogens interact with the endocannabinoid system (29–32). Together, these previous studies suggest a potential interaction between circulating sex hormones and substance use that has implications for depressive symptoms.

Given the link between hormonal cycling and depressive symptoms, one hypothesis is that the use of hormonal birth control (HBC) reduces depressive symptoms by interfering with hormonal cycling. The potential neuroprotective properties of progesterone (33, 34) may contribute to the protective factor of HBC. Although the role of progesterone is still debated (35), studies have shown that metabolites of progesterone, particularly allopregnanolone, may be associated with the severity of depressive symptoms (36–38). Recent studies have also reported a link between neuro-inflammation and depression (39, 40). Progesterone has been shown to have anti-inflammatory properties (41). For example, Gallagher et al. (42) found that females have a longer length of recovery after concussion than males despite the same peak symptom severity. Among females, hormonal contraceptive use was associated with lower symptom severity. The explanation for this finding put forward by Wunderle et al. (43) is that women on hormonal contraceptives who have regulated progesterone levels are protected from the sudden drop in progesterone, providing them some protection against the inflammatory response from head injury. Taken together, previous studies motivate the examination of the effect of HBC use on depressive symptoms.

Given that changes in hormones have been associated with depression and substance use, it may be expected that HBC, which disrupts the cycling of sex hormones, will result in reductions in depressive symptoms. However, results from previous studies examining this relationship have been mixed. For example, a study examining the Finnish population-based Health 2000 study data found that the longer the HBC use the lower the risk of major depressive disorder (44) supporting the hypothesis that HBC use reduces depressive symptoms. However, other studies have found the reverse with a study examining the data from the National Prescription Register and the Psychiatric Central Research Register in Denmark reporting HBC use, particularly in adolescents, resulted in increased use of antidepressants (45). The differences in participant demographics (e.g., age, substance use history) may contribute to differences across studies. The goal of this preliminary study is explore the relationship between HBC use, substance use (alcohol and CB) and depressive symptoms in a group of emerging adult women.

## Methods

### Participants

Three thousand three hundred twenty females completed the series of survey questionnaires, as part of an introductory psychology course at Indiana University for course credit. The study was conducted in a span of 2 years (2015–2017). Inclusion criteria included being enrolled in the introductory psychology course. Conditional exclusion criterion for the final analysis was reporting a non-binary gender identification or identifying as male. Participants gave informed consent as approved by Indiana University's Institutional Review Board for the protection of human subjects.

### Measures

A computer-based, online survey with questions regarding current medications including birth control was administered. The following assessments were included in the survey:

#### Patient Health Questionnaire (PHQ): Depression

The PHQ was employed for the assessment of panic disorder, other anxiety disorders, and depressive disorders (46–48). Depression was assessed using the depression module of the PHQ [PHQ-9; (49)]. Each of the nine PHQ-9 depression items describes one symptom corresponding to one of the nine Diagnostic and Statistical Manual of Mental Disorders, Fourth Edition diagnostic. The scoring protocol used was the one prescribed by the assessment. The score range is zero to 27. There were three classifications: major depressive syndrome, other depressive syndrome, none. For both major and other depressive syndrome, participants had to state that they have “little interest or pleasure in doing things” or “feeling down, depressed, or hopeless” more than half of the days in the past 2 weeks. In addition, for major depressive syndrome an additional five or more questions had to be answered in this manner and for other depressive syndrome 2–4 questions.

#### Alcohol Use Disorders Identification Test (AUDIT)

The AUDIT (50) is a 10-item screening tool developed by the World Health Organization (WHO) to assess alcohol consumption, drinking behaviors, and alcohol-related problems. The scores range from zero to 40. The scores are divided into three categories—safe, hazardous and dependent. A score of eight or more indicate hazardous drinking and a score of 13 or more in women (15 or more in men) indicate possible alcohol use disorder (dependent).

#### Cannabis Use Disorders Identification Test-Revised (CUDIT)

The CUDIT is a brief, 8-item screening measure (51). It is a valid measure for the identification of *likely* cases of DSM-5 cannabis use disorder (CUD) and is a screening tool to identify problematic CB use. The scores range from zero to 32. The scores are divided into three categories—safe, hazardous, and disordered. A score of eight or more indicates hazardous use and 12 or more indicate possible CUD.

### Statistical Analysis

A between subjects ANOVA with PHQ-9 score as the dependent variable with birth control use, CUDIT category, AUDIT category and was performed using SAS version 9.4. Planned *t*-test were performed to examine simple effects using the Satterwaite approximation. Participants with missing data cells were removed for a given analysis.

## Results

### Demographics

The 998 participants who reported using some form of hormonal birth control used a variety of oral contraceptives including progestin only and combination estrogen/progestin pills (see Table 1 for demographic data); 132 of these participants did not know the type or brand of oral contraception they used. In addition, a small percentage used other forms of hormonal birth control −7 used NuvaRing, 11 Depo-Provera shots, and 3 IUDs.

**Table 1:** Demographics.

	**No HBC**	**HBC**	***F***	**N noHBC/HBC**
Age (years)	19.05 ± 1.8	19.0 ± 1.6	<1	2,322/998
Depression-PHQ-9	1.6 ± 2.2	1.3 ± 2.1	11.57^**^	2,322/998
Alcohol-AUDIT	4.6 ± 4.9	5.6 ± 4.9	24.72^**^	2,322/998
CB-CUDIT	2.0 ± 4.1	2.4 ± 3.7	4.96^*^	2,322/998
Race (#)				
White	1552	875		
Black	157	26		
Hispanic	79	10		
Asian	330	11		
Other + No response	204	76		

*HBC, hormonal birth control.*

** *p < 0.001;*

* *p < 0.05*.

### Group Differences

**Figure 1:**
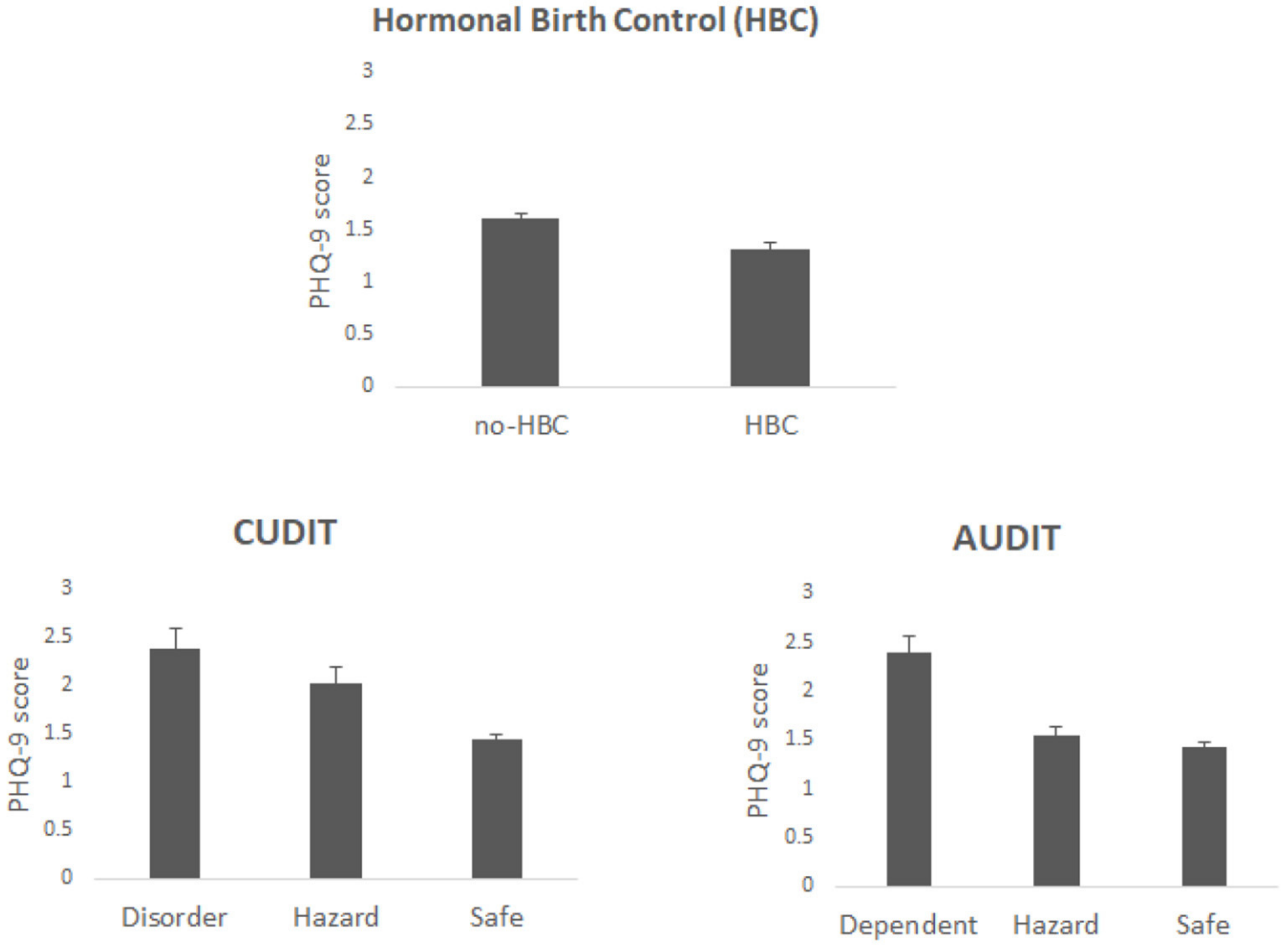
Shows the PHQ-9 scores for each independent variable.

The group demographics are found in Table 1. The results revealed that individuals taking HBC had lower overall PHQ-9 scores than did those not taking HBC [*F*_(1, 3302)_ = 12.45, *p* = 0.0004, η^2^ = 0.0037], see Figure 1. Also, when examining individuals who were classified by the PHQ-9 as having depressive symptoms (i.e., classified with either major or other depressive symptoms), 15.2% (9.9% classified as major depression) of non-HBC users had depressive symptoms while 12.1% (7.6% classified as major depression) of those in the birth control group had depressive symptoms.

**Figure 2:**
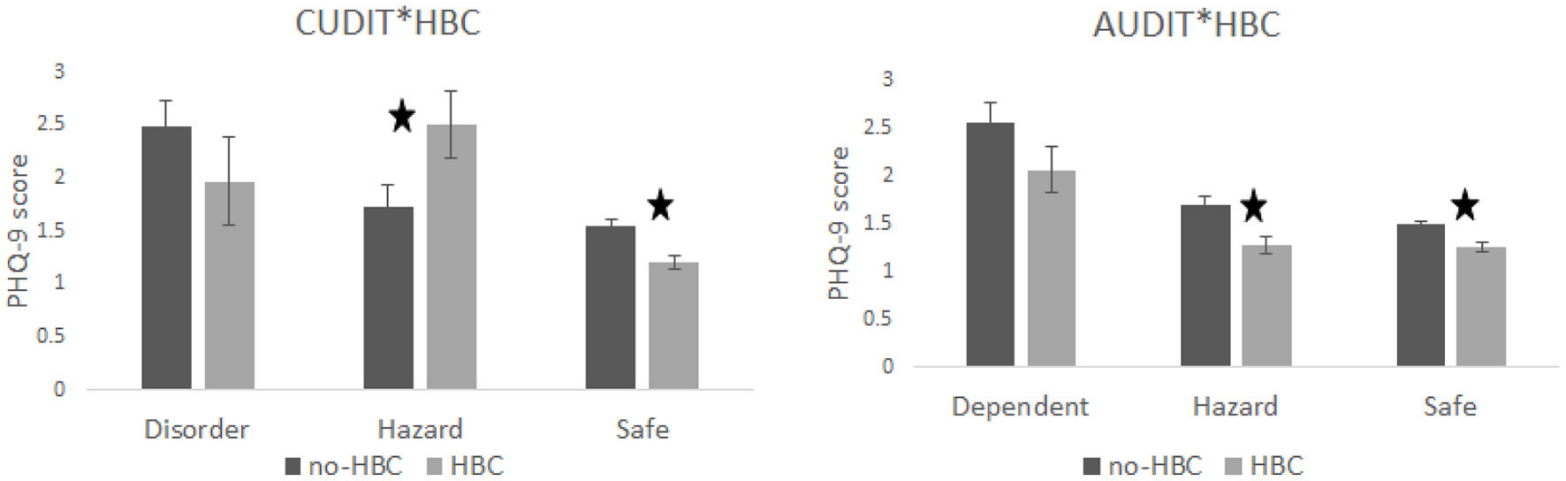
Shows the simple effects. For CUDIT group differences in PHQ-9 scores were observed for the safe and hazard groups with the safe group showing that non-HBC users had higher scores than HBC users. The opposite was true for the CUDIT hazard category, which led to a cross-over interaction. For AUDIT, the safe and hazard group showed significant effects with the non-HBC group having higher PHQ-9 scores than users.

PHQ-9 score significantly varied with CUDIT classification with it being lowest for individuals classified as safe (with low scores) and highest for those classified as disordered (highest scores) [*F*_(2, 3302)_ = 16.97, *p* <0.001, η^2^ = 0.01], see Figure 1. A similar finding was observed for AUDIT [*F*_(2, 3302)_ = 16.31, *p* < 0.001, η^2^ = 0.0096]. Additionally, there was an interaction between HBC use and CUDIT [*F*_(2, 3302)_ = 6.02, *p* = 0.0025, η^2^ = 0.0035]. Planned contrasts showed that for the CUDIT safe group PHQ-9 score was higher for non-HBC user than users (*t* = 4.3, *p* < 0.0001) but for the hazard group the reverse was found (*t* = 2.06, *p* = 0.041) see Figure 2; there was no significant difference for the disordered group (*t* = 1.09, *p* = 0.28). The interaction between HBC use and AUDIT was not significant (*F* <1; planned contrasts: safe—*t* = 2.66, *p* = 0.0079; hazard—*t* = 2.61, *p* = 0.0094; dependent—*t* = 1.42, *p* = 0.16). The 3-way interaction between HBC use, CUDIT and AUDIT was also significant [*F*_(4, 3302)_ = 3.24, *p* = 0.012, η^2^ = 0.0038].

Additionally, HBC users had higher scores on the AUDIT and CUDIT [AUDIT: *F*_(1, 3319)_ = 25.15, *p* < 0.0001, η^2^ = 0.0075; CUDIT: *F*_(1, 3319)_ = 4.71, *p* = 0.03; η^2^ = 0.0014]. In fact, the reported frequency of CB use as indicated on the CUDIT was higher [*F*_(1, 3319)_ = 21.39, *p* < 0.0001; η^2^ = 0.0064] for the HBC user group.

## Discussion

The primary goal of this preliminary study was to explore the relationship between HBC use, substance use and depression in emerging adult women. The results do show a relationship such that those who take HBC have lower PHQ-9 scores and fewer individuals with scores categorized as major depressive symptoms than those who do not take HBC. Substance use also varied with PHQ-9 score such that individuals with high PHQ-9 scores also had higher AUDIT and CUDIT scores. However, it appears that only CB use interacted with HBC use. The results indicate that the CUDIT safe group shows that individuals using HBC have lower depression scores than the non-HBC group, the hazard group showed the reverse with the HBC users having higher PHQ-9 scores; there was no significant difference for the disorder group.

The relationship between depression and sex hormones is a complex one. The current study suggests that taking HBC reduces depressive symptoms as measured by the PHQ-9, at least in emerging adult women. Previous studies have found similar effects (44, 52). However, other studies have failed to find such a benefit in the use of HBC (45). One explanation is that individuals who do not tolerate HBC well, likely due to the negative impact on mood, stop its use. Those individuals may have a progesterone intolerance, which may cause these adverse effects on mood (53). Support for this explanation can be found in a recent study in which 16 year olds were found to show an adverse relationship between HBC use and depression; however, this relationship was not found at older ages (54) suggesting that those 16 year olds with adverse reactions stopped taking HBC or adapted to it.

Several studies have shown a relationship between substance use, both alcohol and CB, and depression (21). In fact, recent studies have shown an increase in use of both substances during the premenstrual phase when progesterone is rapidly decreasing (27, 28). This phase of the menstrual cycle has also been linked to increased depressive symptoms (17). Together this suggests a potential relationship between sex hormones, particularly progesterone, depression and substance use. The results presented in the current study provide further support for these previous findings in that alcohol, CB and HBC use all predicted PHQ-9 with increased substance use correlating with increased depressive symptoms. It should be noted that this increase in symptoms with increasing use is in spite of finding that women on HBC have higher AUDIT and CUDIT scores and they have lower PHQ-9 scores.

Interestingly, the relationship between HBC and depression does not appear to be affected by alcohol use. Individuals using HBC had lower PHQ-9 scores regardless of AUDIT category, although for the disordered group the difference was not significant. Progesterone has been found to have neuroprotective properties (33, 34). Recently it has been suggested that progesterone reduces neuroinflammation (55). Alcohol has been shown to cause neuroinflammation (41) and damage to regions related to emotional regulation (56). As a result, the stabilizing effect of progesterone in hormonal birth control may potentially have the side effect of reducing the impact of alcohol use on depression by reducing the inflammatory effect of alcohol. Further research is necessary to test this hypothesis.

The relationship between CB use and HBC was different from that between alcohol use and HBC. Individuals classified as hazard users who also use HBC had significantly higher PHQ-9 scores than hazardous CB users who did not use HBC. The underlying mechanism that accounts for this interaction is unknown. However, CB and alcohol have different mechanisms of action on brain and potentially interact differently with sex hormones. In a clinical trial, progesterone was found to reduce cannabis withdrawal symptoms in individuals with cannabis use disorder (57). Pre-clinical studies have found that estrogens interact with the endocannabinoid system. For example, estradiol regulates cannabinoid receptor density (58), transcription (30) and signal transduction (32). Additionally, the endocannabinoid system is affected by hormonal cycling (29). Also, there are several studies that link depression and CB use; with many individuals with depression, anxiety and psychosis using CB to self-medicate (59, 60). This may be associated with a relationship between regions linked to emotional regulation such as the medial prefrontal cortex and the hippocampus, both of which have a high density of CB1 receptors potentially making them the locus of the relationship between CB and depression. Furthermore, CB has not been found to cause neuroinflammation but instead may help to reduce it (61). Again, the current study suggests that the mechanism of action for CB is different from that of alcohol.

## Limitations

The results presented should be interpreted with some caution, as there are limitations to the study. First, it may be difficult to collect sensitive data such as questions about substance use, particularly CB due to it being illegal in many states. However, the online nature of the study was expected to increase compliance. Second, the study is correlational; therefore, no causal inferences can be made. The study is designed to help direct future research and the associations observed have done just that. Third, this is a between subjects design and there could be other, uncontrolled, differences between groups. As mentioned above, individuals who do not tolerate HBC due to its effect on mood may discontinue use. Therefore, our birth control group is likely biased. Also, given that the women on birth control also have higher AUDIT and CUDIT scores, there are likely personality differences between groups that were not controlled. These two possibilities of sources of groups differences are in addition to potential cultural differences (e.g., progressive vs. traditional) which may make some behaviors more socially accepted. Another limitation of the study is a lack of detailed information about a history of contraception use and premenstrual dysphoric disorder both of which may influence results. A final major limitation is that the assessment of depression and substance use was performed using self-report surveys and not in-depth interviews. Therefore, the categorizations used are not clinical ones. Although there are a number of limitations of this preliminary study that make it difficult to determine the mechanism by which HBC may interact with depressive symptoms or substance use, the results do indicate that there is indeed a relationship between these factors. The results also guide future research and strongly support longitudinal, within-subject studies to systematically examine the relationships between substance use, hormonal birth control, and depression.

## Conclusions

The current study found that those females taking HBC have lower PHQ-9 scores, fewer depressive symptoms, and have higher AUDIT and CUDIT scores. Additionally, the results replicate previous findings in that alcohol and CB use were both found to be associated with PHQ-9 score. Finally, we report that CB use may interact with HBC differently than alcohol use. Together the results presented provide significant motivation to further explore the relationship between CB use, depression and circulating sex hormones in females.

## Data Availability Statement

The raw data supporting the conclusions of this article will be made available by the authors, without undue reservation.

## Ethics Statement

The studies involving human participants were reviewed and approved by Indiana University. The patients/participants provided their written informed consent to participate in this study.

## Author Contributions

The author confirms being the sole contributor of this work and has approved it for publication.

## Funding

This publication was funded by the Indiana Clinical and Translational Sciences Institute, funded in part by grant # UL1TR002529 from the National Institutes of Health, National Center for Advancing Translational Sciences, Clinical and Translational Sciences Award.

## Author Disclaimer

The content is solely the responsibility of the authors and does not necessarily represent the official views of the National Institutes of Health.

## Conflict of Interest

The author declares that the research was conducted in the absence of any commercial or financial relationships that could be construed as a potential conflict of interest.

## Publisher's Note

All claims expressed in this article are solely those of the authors and do not necessarily represent those of their affiliated organizations, or those of the publisher, the editors and the reviewers. Any product that may be evaluated in this article, or claim that may be made by its manufacturer, is not guaranteed or endorsed by the publisher.
