# Supplementary material for: Association Between Hormonal Birth Control, Substance Use, and Depression
Source: Front Psychiatry. 2022 Feb 8;13:772412. doi: 10.3389/fpsyt.2022.772412 (PMC8861494; doi:10.3389/fpsyt.2022.772412)
Supplement: Supplementary file 1 [file Data_Sheet_1.PDF]

Supplementary Materials

Linear Regression Analysis with PHQ-9 as dependent variable.

Root MSE	2.15439	R-Square	0.0234
Dependent Mean	1.52028	Adj R-Sq	0.0220
Coeff Var	141.71021		

Parameter Estimates

Variable	DF	Parameter Estimate	Standard Error	t Value	Pr > |t|
Intercept	1	1.37184	0.06176	22.21	<.0001
HBC use	1	-0.30538	0.12189	-2.51	0.0123
CUDIT	1	0.04889	0.01181	4.14	<.0001
AUDIT	1	0.02974	0.00990	3.00	0.0027
HBC*CUDIT	1	0.03866	0.02313	1.67	0.0947
HBC*AUDIT	1	-0.02146	0.01795	-1.20	0.2320

Correlation Analysis

Pearson Correlation Coefficients
Prob > |r| under H0: Rho=0
Number of Observations

	PHQ-9	CUDIT	AUDIT
PHQ-9	1.00000	0.12575<br><.0001	0.08752<br><.0001
CUDIT	0.12575<br><.0001	1.00000	0.37955<br><.0001
AUDIT	0.08752<br><.0001	0.37955<br><.0001	1.00000
